# SZ-685C inhibits the growth of non-functioning pituitary adenoma by down-regulating miR-340-3p and inducing autophagy

**DOI:** 10.1016/j.heliyon.2024.e37230

**Published:** 2024-08-30

**Authors:** Xin Wang, Zhong-Yu Wang, Hui-Tong Chen, Yu-You Luo, Si-Yuan Li, Xiong-Ming Luo, Jun-Hua Yang, Yu-Xin Ma, Xiao-Bao Jin, Jing Liu, Zong-Ming Wang

**Affiliations:** a Department of Human Anatomy, Histology and Embryology, School of Basic Medical Sciences, Guangdong Pharmaceutical University, Guangzhou, 510006, China; b Pituitary Tumor Center, Department of Neurosurgery, The First Affiliated Hospital, Sun Yat-sen University, Guangzhou, 510080, China; c School of Life Sciences and Biopharmaceutics, Guangdong Pharmaceutical University, Guangzhou, 510006, China; d Guangdong Provincial Key Laboratory of Pharmaceutical Bioactive Substances, Guangdong Pharmaceutical University, Guangzhou, 510006, China

**Keywords:** PDFS, SZ-685C, Non-functioning pituitary adenoma, Autophagy, miR-340-3p

## Abstract

**Background:**

SZ-685C, an anthracycline compound derived from the mangrove endophytic fungus *Halorosellinia* sp. (No. 1403) collected from the South China Sea, has shown strong anticancer activities. Non-functioning pituitary adenomas (NFPAs) are a type of tumor that can be challenging to manage clinically and have a significant unmet medical need. Our research has found that SZ-685C showed an inhibitory effect on the viability, migration ability, and proliferation ability of a human non-functioning pituitary tumor-derived folliculostellate (PDFS) cell line.

**Methods:**

SZ-685C was prepared and purified from the mangrove endophytic fungus No. 1403. PDFS cells were exposed to SZ-685C, and the effect of SZ-685C on PDFS cells was evaluated. RNA sequencing was used to analyze the miRNA expression profile in PDFS cells of the control group and SZ-685C-treated group. Quantitative polymerase chain reaction (qPCR) was performed to verify the expression of selected miR-340-3p. The effects of SZ-685C on PDFS cells after overexpression of miR-340-3p were evaluated. Dual-luciferase reporter assays showed PPP1CB is a direct target of miR-340-3p. Finally, the action pathway of the selected miR-340-3p was predicted and evaluated through bioinformatics analysis.

**Results:**

SZ-685C reduced cell viability in PDFS cells, accompanied by inhibition of migration ability and proliferation ability. The IC50 value for 24 h is 9.144 ± 0.991 μM, and for 48 h is 4.635 ± 0.551 μM. SZ-685C increased the protein levels of Beclin 1, the ratio of LC3-II to LC3-I, and LAMP-1, and down-regulated p62. MiRNA sequencing and further validation showed that miR-340-3p significantly decreased in PDFS cells treated with SZ-685C. After overexpression of miR-340-3p, the inhibition of viability, migration ability, proliferation ability, and autophagy-promoting effect of SZ-685C on PDFS cells were weakened. SZ-685C caused a decrease in PPP1CB expression and activation of the ERK pathway in PDFS cells, and this trend was reversed after overexpression of miR-340-3p.

**Conclusions:**

SZ-685C downregulates the expression of miR-340-3p in PDFS cells, thereby reducing the expression of PPP1CB and activating the ERK pathway to promote autophagic cell death, leading to inhibition of PDFS cell growth.

## 1 Introduction

SZ-685C is an anthraquinone derivative derived from cultures of the mangrove endophytic fungus Halorosellinia sp. (No. 1403) collected from the South China Sea [1]. Several earlier studies indicated that it is a potential anti-cancer drug candidate. SZ-685C was discovered to suppress the proliferation of various cancer cell lines and have direct apoptosis induction through external and internal apoptosis pathways [2]. Moreover, SZ-685C was found to override adriamycin-resistance and radioresistance in breast cancer cells and nasopharyngeal carcinoma cells respectively [3,4]. It was shown that SZ-685C possessed a good prospect for development of cancer drugs.

Pituitary adenoma (PA) ranks as the second most prevalent CNS tumor in terms of histology, and it is more prevalent in patients between the age of 14 and 39 [5]. Non-functioning pituitary adenomas (NFPA) account for a large part of them, about 15 %–30 %. Non-specific headache, visual impairment, sporadic pituitary apoplexy, and other mass effect-related symptoms and signs are typically used to diagnose it [6]. Due to the lack of clear criteria, it is more challenging to manage and follow up than other functional hormone-secreting pituitary adenomas [7]. Surgery is usually the first-line treatment of NFPA, but the risk of pituitary function deterioration after surgery is up to 10 % [6]. For patients who cannot tolerate surgery, nontraumatic treatment may be more appropriate for incidentally found pituitary adenomas. Dopamine agonists (DA) and somatostatin synthetic analogs (SSA) may be used to control NFPA after all other options have failed, but it is still necessary to create effective anti-NFPA medications [8]. In our previous study, we confirmed that SZ-685C induced apoptosis in primary human non-functioning pituitary adenoma cells and rat prolactinoma cell line, MMQ cells [9,10]. And SZ-685C induced cell cycle arrest at the G2/M phase in CNE2 cells (human nasopharyngeal carcinoma cell line) and CNE2R cells (stable radioresistant nasopharyngeal carcinoma cell line) [4]. The anti-tumor effect of SZ-685C is well illustrated.

In many cancers, autophagy—a form of programmed cell death—is pervasive. Overactivation of autophagy can result in autophagic cell death, thereby inhibiting tumor progression [11]. Autophagy can maintain the stability of the genome and prevent the accumulation of p62 proteins which are carcinogenic, thus preventing the occurrence, proliferation, invasion, and metastasis of tumors. Consequently, autophagy contributes to the process of tumor suppression, particularly in the early stages of tumor occurrence [12]. For instance, Cucurbitacin I induces pro-death autophagy in A549 cells by activating the ERK signaling pathway [13].

More and more studies show that non-coding RNA (ncRNA) is involved in multi-level regulation of gene expression in vivo, and plays a key role in the core process of molecular biology by regulating cell differentiation and development [14]. The most commonly noticed and first discovered ncRNAs are microRNAs (miRNAs), which are composed of 18–30 nucleotides and play a crucial role in post transcriptional gene regulation in eukaryotes by regulating its targets [15,16]. It has been clinically proven that miRNA mediated therapy combined with other therapies has good application prospects in tumor treatment. For example, some anti-tumor drugs can manipulate the biogenesis of miRNAs, which is closely related to the drug therapy response of tumors [17]. MiRNA can also serve as a prognostic biomarker in tumor treatment, guiding the clinical application of anti-tumor drugs [18]. In breast cancer, lung cancer, colon cancer, gastric cancer and ovarian cancer, miRNA has been explored as a potential biomarker and target [19].

MiRNA plays an important regulatory role in autophagy during the chemical resistance of tumors to drugs. MiRNA can alter the levels of key proteins in different steps of the autophagy pathway, including the entire process from upstream signaling pathway activation stage to lysosomal degradation stage [20]. In different diseases, Beclin1 may be negatively regulated by multiple miRNAs such as miR-376b, miR-30a, and miR-216a, leading to a decrease in autophagy activity [21]. Previous studies have found that SZ-685C can significantly inhibit MMQ cell proliferation by downregulating miR-200c [9], and whether its mechanism of action is related to regulating autophagy pathways remains to be further explored.

In this study, we sought to explore whether SZ-685C could control cell proliferation and migration, and induce autophagic cell death in PDFS, and whether this effect is achieved by regulating miRNA and its downstream molecules.

## 2 Materials and methods

### 2.1 Chemicals and reagents

**Fig. 1:**
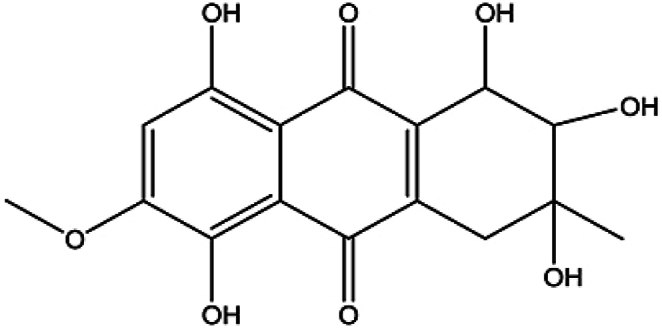
Chemical structure of SZ-685C.

SZ-685C was produced and purified from mangrove endophytic fungus Halorosellinia sp. (No. 1403) [2]. Its structure was identified by interpretation of spectral data (MS, 1H NMR, 13C NMR, 2D NMR) and X-ray single crystal diffractive technique (Fig. 1). As a stock solution, the substance was dissolved in 0.5 % DMSO at a concentration of 1 mM. And the DMSO content of the control group was consistent with the SZ-685C treatment group. SZ-685C (98 % purity as determined by HPLC) was obtained from Guangdong Province Key Laboratory of Functional Molecules in Oceanic Microorganism (Sun Yat-sen University).

### 2.2 Cell culture

The human pituitary tumor-derived folliculostellate cell line, PDFS, were originated from a clinically non-functioning pituitary macroadenoma [21]. The human liver cell lines, L-O2, were sourced from the American Type Culture Collection (ATCC). Cells were cultured in DMEM medium (Gibco, USA) supplemented with 10 % (v/v) fetal bovine serum (FBS) and 1 % penicillin-streptomycin at 37 °C in a humidified atmosphere of 5 % CO_2_.

### 2.3 Cell viability assay

Cell viability was determined by Cell Counting Kit-8 assay (CCK-8, Biosharp, China). Cells harvested in the exponential growth phase were seeded into 96 well-plates with a 100 μL medium at a density of 1 × 10^4^ cells/well. After 24 h of culture, cells were treated for another 24 h with diluted concentrations of SZ-685C, and then 10 μL CCK-8 reagent was added into each well. After incubating at 37 °C for 1 h, the absorbance of each well was measured at 450 nm on a microplate reader (Bio-rad, USA). The half-maximal inhibitory concentration (IC_50_) was estimated.

### 2.4 Wound healing assay

PDFS cells were seeded on 6 well-plates at a density of 1 × 10^6^ cells/well and cultured overnight without FBS. The cells were scratched with the pipette tip and were gently washed once with PBS. The cells were then treated with IC_50_ of SZ-685C and incubated for 24 h in the medium. Images were captured at 0 h and 24 h of drug treatment. The cell morphology was observed under an inverted microscope (Optec, China). Three fields in each well were photographed.

### 2.5 Colony formation assay

PDFS cells that were previously treated with IC_50_ of SZ-685C for 24 h were cultured in 6-well plates (1000 cells/well). After 11 days, the colonies were fixed by polymethylene and stained with 0.1 % crystal violet for 20 min. The colonies that contained more than 50 cells were counted and photographed.

### 2.6 Immunofluorescence assay

PDFS cells were seeded on 6 well-plates at a density of 5 × 10^5^ cells/well and cultured overnight. The cells were then treated with IC_50_ of SZ-685C and incubated for 24 h in the medium. Then, cells were fixed by polymethylene for 30 min, washed with PBS three times, and treated with primary and secondary antibodies (rabbit anti-LC3B 1:200, rabbit anti-Beclin1 1:200, rabbit anti-p62 1:200, rabbit anti-LAMP-1 1:200; Dyligh 488-goat anti-rabbit IgG 1:400). The cells were stained with Hoechst 33342 at 37 °C for 30 min. The cell morphology was observed under a fluorescence microscope (Leica, Germany). Three fields in each well were photographed.

### 2.7 Western blot assay

PDFS cells (2 × 10^6^) cultured in 6 well-plates were treated with IC_50_ of SZ-685C for 24 h. Cells were then lysed in RIPA lysis buffer containing 1 % phosphatase inhibitor and 1 % protease inhibitor cocktail (Meilunbio, China) for 30 min, and the protein concentrations in the cleared supernatants were gauged using the bicinchoninic acid (BCA) protein assay. The cell proteins were separated by SDS-PAGE and transferred to polyvinylidene difluoride (PVDF) membrane (Immobilon, Ireland), followed by blocking with 5 % skim milk in TBS-T and treated with primary and secondary antibodies (rabbit anti-LC3B 1:1000, rabbit anti-p62 1:1000, rabbit anti-Beclin 1 1:1000, rabbit anti-LAMP-1 1:1000, rabbit anti-p-ERK1/2 1:1000, rabbit anti-ERK1/2 1:1000, mouse anti-β-Tubulin 1:1000; HRP goat anti-rabbit IgG 1:10000, HRP goat anti-mouse IgG 1:6250). Membranes were then washed three times with TBS-T and detected by electrochemiluminescence (ECL) detection system according to the manufacturer's instructions.

### 2.8 Extraction of total RNA

PDFS cells (2 × 10^6^) cultured in 6 well-plates were treated with IC_50_ of SZ-685C for 24 h. Cells were then treated in TRIzol (Ambion, USA) for 15 min. Then the liquid was collected into the EP tubes, treated with chloroform, shook and placed for 10 min. After centrifugate, the colorless water layer were transferred to the new EP tubes and the RNA were precipitated with isopropanol and then washed with anhydrous ethanol. The RNA was dried, added RNA free water to fully dissolve and mix. The purity and concentration of RNA were determined by ultramicro spectrophotometer.

### 2.9 RNA sequencing and analysis

Cell samples were tested by Beijing Nuohe Zhiyuan Technology Co., Ltd. Nanodrop detection, agarose gel electrophoresis analysis and Qubit precise quantitative Agilent 2100 were used to accurately detect total RNA samples. A library was built according to the instructions for using Small RNA Sample Pre Kit. Qubit 2.0 was used to preliminarily quantify the cDNA library and dilute it to 1 ng/μL. Accurately its effective concentration using qPCR experiments was quantified. After pooling the different libraries that have passed the library check according to the requirements, using Illumina SE50 was used to sequence them.

After length screening, locate the sRNA on the reference sequence, analyze the distribution of small RNA on the reference sequence, map the above distribution to the reads on the reference sequence, and compare it with the specified range sequence in miRBase to obtain detailed information on the sRNA on each sample matching, including the secondary structure of known miRNAs on the matching, the sequence, length, and frequency of occurrence of miRNAs in each sample. The input data for miRNA differential expression is the readcount data obtained from miRNA expression level analysis. For samples with biological duplication, we use DESeq2 based on negative binomial distribution for analysis.

MiRDB (http://www.mirdb.org/) predicted the target mRNA of known miRNAs with the most significant differential expression. Using the TargetScan database (https://www.targetscan.org/) Analyze the binding sites between miRNA and the target. KEGG pathway enrichment analysis uses KEGG Pathways as a unit and applies hypergeometric tests to identify Pathways that are significantly enriched in candidate target genes compared to the entire genome background. Protein Interaction Network Analysis (https://cn.string-db.org/) was used to predict the interacting proteins of the target.

### 2.10 Reverse transcription fluorescence quantitative polymerase chain reaction (qRT-PCR)

After the sample is qualified, the system can be prepared according to the corresponding instructions of the reagent kit (20 μL) Perform reverse transcription reaction to obtain cDNA samples from each group. The reverse transcription of miRNA requires the miRNA 1st Strand cDNA Synthesis Kit (by stem-loop) method, while the reverse transcription of other genes requires the Hifair III 1st Strand cDNA Synthesis SuperMix for qPCR method. Design the sequence of detection wells, take eight tubes, operate on ice, and prepare the system according to the instructions of the ChamQ Universal SYBR qPCR Master Mix reagent kit (20 μL). Each group is equipped with 3 secondary holes. The gene expression levels of each group were calculated using the 2^-△Ct^ method. The expression level of U6 gene was used as an internal control. ΔCt = Ct _testing gene_ - Ct _internal control_.

hsa-miR-340-3p RT primer:

5′-CTCAACTGGTGTCGTGGAGTCGGCAATTCAGTTGAGATAAAG-3'.

hsa-miR-340-3p forward primer: 5′-CCGTCTCAGTTACTTTAT-3'.

hsa-miR-340-3p reverse primer: 5′-AGTCGGCAATTCAGTT-3'.

hsa-U6 forward primer: 5′-ATACAGAGAAAGTTAGCACGG-3'.

hsa-U6 reverse primer: 5′-GGAATGCTTCAAAGAGTTGTG-3'.

### 2.11 Cell transfection assay

PDFS cells (2 × 10^6^) cultured in 6 well-plates were spread and incubated overnight. DMEM culture media containing mimic-NC and hsa-miR-340-3p mimic (transfection concentration 10 nM) were prepared respectively. The prepared culture medium was added to each well and incubated in a 37 °C incubator for 48 h, followed up with SZ-685C treatment for 24 h according to experimental requirements.

hsa-miR-340-3p NC:

sense 5′-UUGUACUACACAAAAGUACUG-3'.

antisense 5′-GUACUUUUGUGUAGUACAAUU-3'.

hsa-miR-340-3p mimic:

sense 5′-UCCGUCUCAGUUACUUUAUAGC-3'.

antisense 5′-UAUAAAGUAACUGAGACGGAUU-3'.

### 2.12 Dual-luciferase reporter assays

PDFS cells were plated in a 96-well plates. Lipofectamine 3000 (Invitrogen) was used to cotransfect the cells with miR-340-3p mimics and a reporter plasmid that has a luciferase gene containing the target sequence of the wild-type (WT) 3′-UTR of PPP1CB and the mutant 3′-UTR of PPP1CB. (Fenhui Biotechnology Co., Ltd, Hunan, China). PDFS cells were collected 48 h after transfection and analyzed using the Dual-Luciferase Reporter Assay System (Promega, E1910). Transfections were performed in triplicate and repeated at least three times in separate experiments.

### 2.13 Statistical analysis

For statistical analysis, the GraphPad Prism software v. 5.0 was used. Data are presented as the mean ± standard error of the mean. Student's *t*-test was employed to assess the statistical significance of differences between a pair of data sets. Single factor ANOVA analysis is used to evaluate the significance of differences between two or more datasets. *P* < 0.05 was considered statistically significant.

## 3 Results

### 3.1 SZ-685C inhibits the proliferation of PDFS, GH3 and MMQ cells

**Fig. 2:**
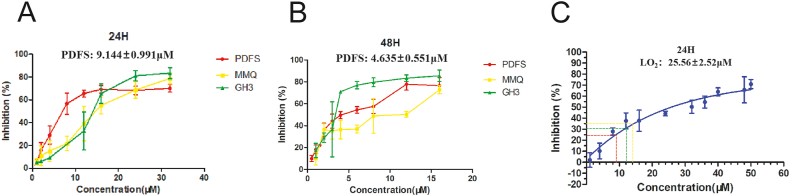
SZ-685C inhibits the growth of PDFS, GH3 and MMQ cells. A shows the inhibition of SZ-685C on PDFS, GH3 and MMQ cells in 24 h; B shows the inhibition of SZ-685C on PDFS, GH3 and MMQ cells in 48 h; C shows the inhibition rate of SZ-685C on L-O2 cells in 24 h; The x coordinate value corresponding to the dashed guideline represents the 24 h IC50 of SZ-685C in PDFS cells.

From the results of the CCK-8 assay, it was found that the viability of the PDFS, GH3 and MMQ cells was suppressed in an SZ-685C dose-dependent manner (Fig. 2A and B). For PDFS cells, the 24 h IC_50_ value is 9.144 ± 0.991 μM and 48 h IC_50_ value is 4.635 ± 0.551 μM. For GH3 cells, the 24 h IC_50_ value is 12.735 ± 2.341 μM and 48 h IC_50_ value is 3.227 ± 0.460 μM. For MMQ cells, the 24 h IC_50_ value is 14.726 ± 0.976 μM and 48 h IC_50_ value is 7.967 ± 0.680 μM. These results suggest that SZ-685C is effective in inhibiting the growth of PDFS cells. In the follow-up experiments, the IC_50_ value of SZ-685C for 24 h was used as the suitable treatment concentration for PDFS cells. What's more, the effect of SZ-685C on the LO2 cell line was examined. As shown in Fig. 2C, the IC_50_ value of SZ-685C in LO2 cells for 24 h was 25.56 ± 2.52 μM. The IC_50_ inhibitory effect of SZ-685C in PDFS cells is about 25 % of that in LO2 cells, indicating that SZ-685C may be relatively safe in vitro.

### 3.2 SZ-685C inhibits the migration and the proliferation of PDFS cells

**Fig. 3:**
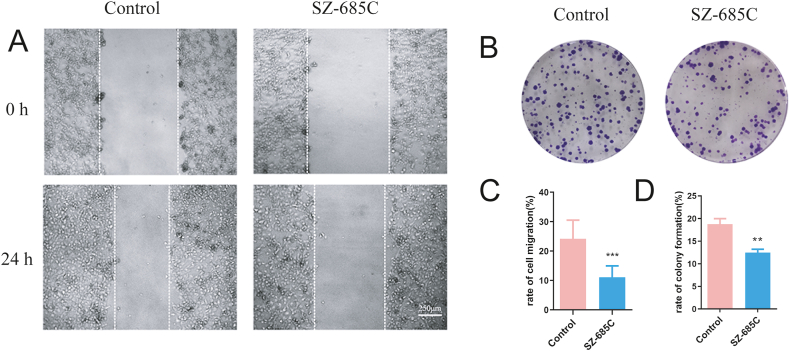
SZ-685C inhibits the migration and proliferation of PDFS cells. A shows the result of wound healing assay and C is the analysis of cell migration rate (%, = the cell migration distance/original distance), scale bar = 250 μm; B shows the result of colony formation assay; each of the colonies contains more than 50 cells was calculated; and D is the analysis of colony formation rate (%, = the number of colonies formed/the number of seeded cells per well). Data are expressed as mean ± standard deviation (analysis of variance, **p < 0.01, ***p < 0.001).

To test whether SZ-685C treatment can curb the migration property of PDFS cells, wound healing assay and colony formation assay were performed. Inhibition of the wound closure after SZ-685C treatment was observed in the micrographs (Fig. 3A), indicating that SZ-685C inhibits the migratory ability of PDFS cells (Fig. 3C). From the results of the colony formation assay, it was found that, compared with the control group, SZ-685C could decrease the number of PDFS cell colonies after 11 days of culture (Fig. 3B–D).

### 3.3 SZ-685C induces the autophagy in PDFS cells

**Fig. 4:**
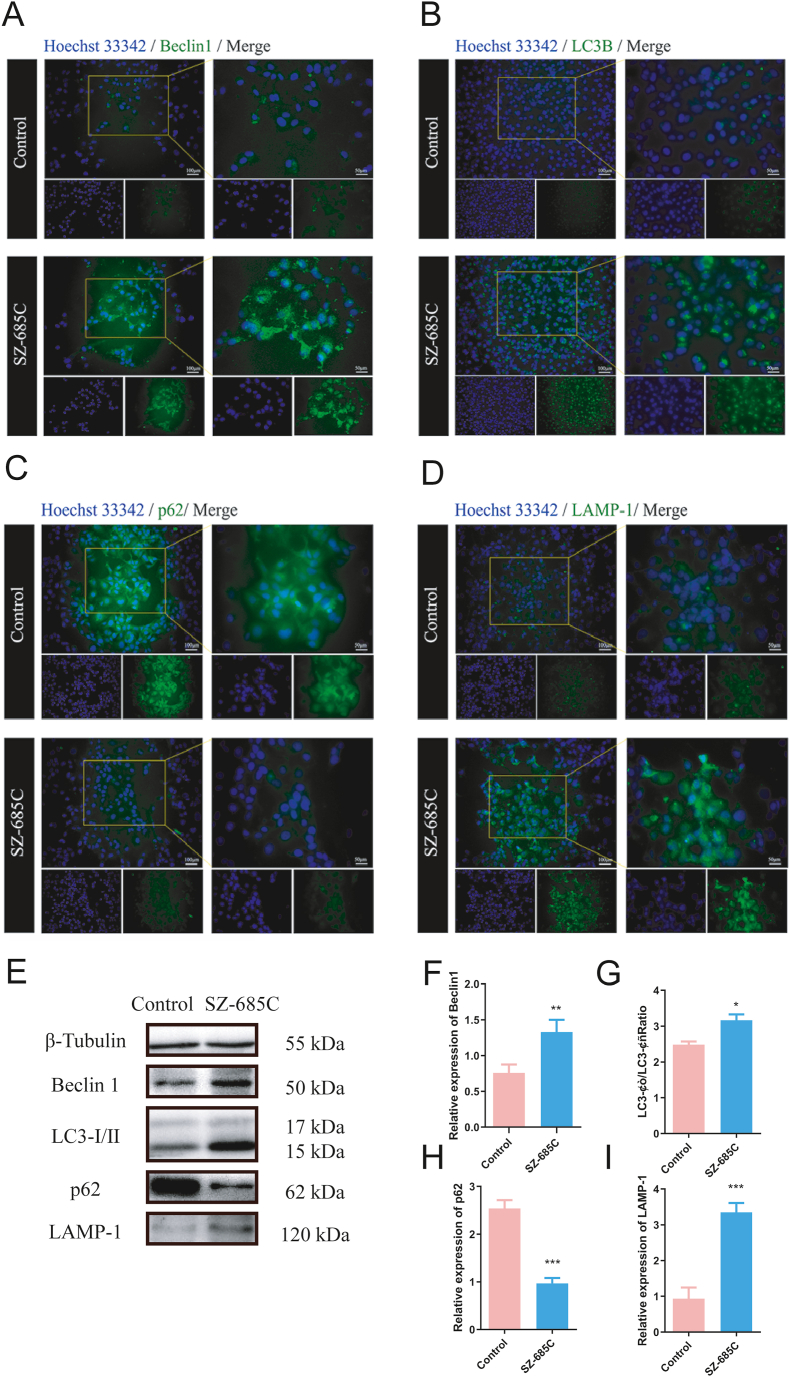
SZ-685C induces autophagy in PDFS cells. A-D show the immunofluorescence results of Beclin1, LC3B, p62 and LAMP-1 protein in PDFS cells, scale bar = 100 μm or 50 μm; E shows the Western blot results of autophagy associated proteins, Beclin1, LC3-Ⅰ, LC3-Ⅱ, p62 and LAMP-1, in PDFS cells (The original image is provided in Supplementary file); F-I are the analysis of E. Data are expressed as mean ± standard deviation (analysis of variance, *p < 0.05, **p < 0.01, ***p < 0.001).

Autophagic cell death is another important mechanism of drug treatment for cancer. To determine if SZ-685C-mediated killing of PDFS cells involves modulation in the expression of autophagic genes, the expression levels of the autophagy-related proteins Beclin1, LC3B (includes LC3-I and LC3-II), p62, and LAMP-1 were quantified through immunofluorescence assay (Fig. 4A–D). It was found that, compared with the control group, SZ-685C significantly up-regulated the expression levels of Beclin1, LC3B, and LAMP-1, and down-regulated the expression of p62 in PDFS cells. Then, Western blot analysis was used to detect other autophagy-related proteins (Fig. 4E). It was found that in the SZ-685C-treated cells, the expression of Beclin1 increased, the ratio of LC3-II to LC3-I rose, the expression of p62 protein decreased, and the expression of LAMP-1 protein increased (Fig. 4F–I). Therefore, it is suggested that SZ-685C-mediated cell death in PDFS cells may involve autophagy.

### 3.4 SZ-685C reduces expression of miR-340-3p in PDFS, MMQ and GH3 cells

**Fig. 5:**
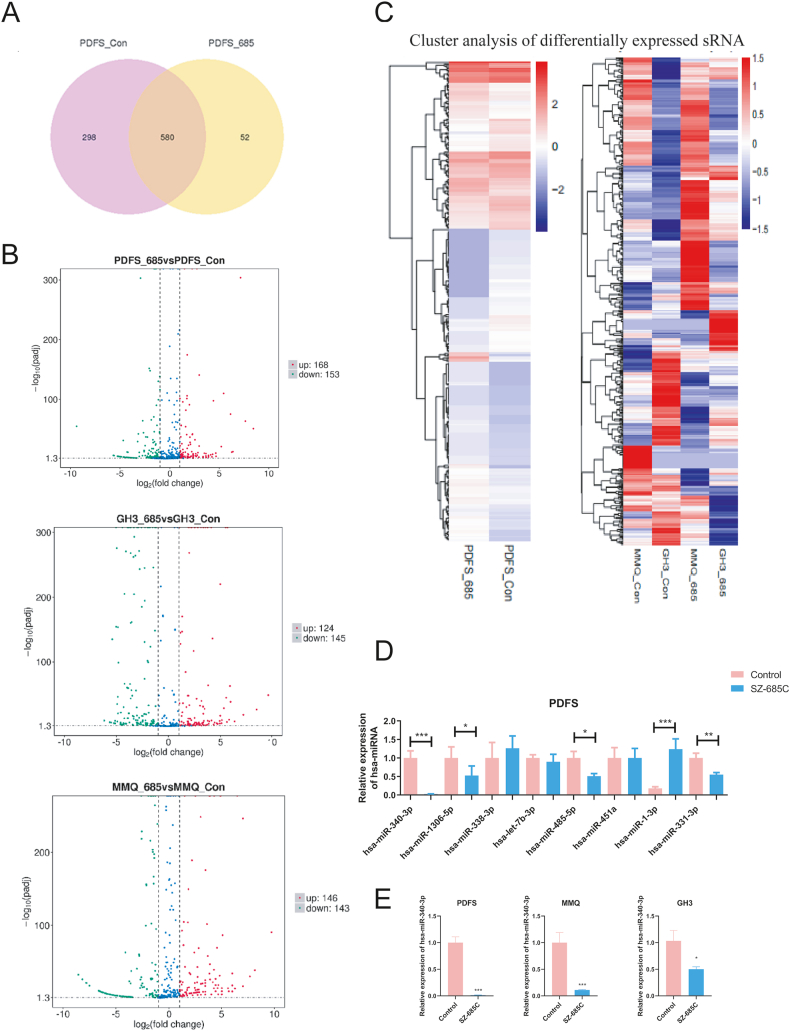
Transcriptome sequencing and the differential miRNAs. A shows the differential miRNA Venn map; B show the differential miRNA volcano map; C shows the differential miRNA cluster diagram; D shows the differential miRNAs detected by qRT-PCR assay. E were the results of qPCR validation. Data is expressed as mean ± standard deviation (analysis of variance, *p < 0.05, **p < 0.01, ***p < 0.001).

To investigate the specific miRNAs that play a regulatory role in the inhibition of cell growth by SZ-685C, total RNA was extracted from PDFS, MMQ and GH3 cells in the SZ-685C treatment group and the control group and the transcriptome was sequenced. Compared to the control group, a total of 168 upregulated miRNAs and 153 downregulated miRNAs were found in PDFS cells treated with SZ-685C (Fig. 5A and B), and the partial differential miRNA results are shown in Table 1. The qPCR experiment was performed to validate the differential miRNAs detected by sequencing (Fig. 5D). We found that miR340-3p was downregulated simultaneously in all three cell lines, so we performed qRT-PCR validation and focused on miR340-3p in the follow-up (Fig. 5E).

**Table 1:** RNA-Sequencing results of common differential miRNAs in PDFS, MMQ and GH3 cells.

sRNA	PDFS_685_readcount vs PDFS_Con_readcount log2FoldChange	MMQ_685_readcount vs MMQ_Con readcount log2FoldChange	GH3_685_readcount vs GH3_Con readcount log2FoldChange
miR-1-3p	7.151456467	3.027740776	9.595157475
miR-451a	2.722620126	4.895889346	1.646538002
miR-338–3p	2.336196347	4.17974387	2.358118278
miR-127–5p	1.962737951	2.442778276	2.550763356
miR-485–5p	1.73829479	4.835095698	4.638226197
miR-138–5p	1.688498091	1.705812681	2.660681048
miR-29c-5p	−1.471158575	−1.687485833	−2.140888345
miR-10a-5p	−1.503617825	−1.633058466	−2.006525067
miR-340-3p	−1.521784648	−6.593613316	−2.284425775
miR-27a-5p	−1.581426355	−4.102829787	−4.835817697
miR-27b-5p	−1.602403109	−5.33530132	−3.149676362
miR-1306–5p	−3.668853435	−4.617072288	−2.684453106
let-7b-3p	−5.594852854	−4.617072288	−4.289340149

### 3.5 The inhibitory effect of SZ-685C on PDFS cells weakened after overexpression of miR-340-3p

**Fig. 6:**
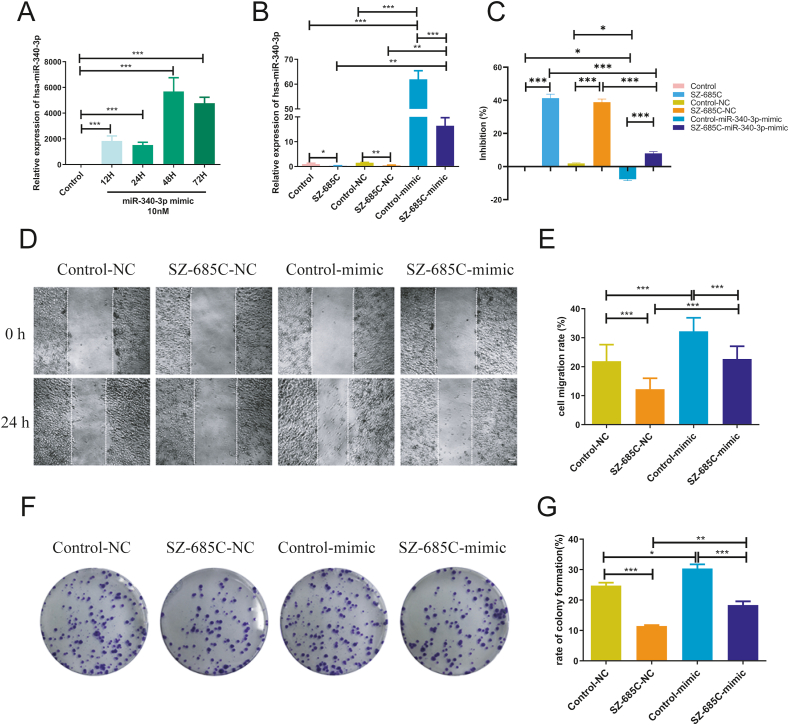
The effect of SZ-685C on the viability, migration and proliferation of PDFS cells after miR-340-3p mimic transfection. A shows expression of miR-340-3p in PDFS cells after transfection of 12, 24, 48 and 72 h; B shows the expression of miR-340-3p in cells of each group after transfection of 48 h; C shows the effect of SZ-685C on the viability of PDFS cells after miR-340-3p mimic 48 h transfection; D shows the effect of SZ-685C on the migration ability of PDFS cells after miR-340-3p mimic 48 h transfection, scale bar = 250 μm, and E is the analysis of D; F shows the effect of SZ-685C on the proliferation ability of PDFS cells after miR-340-3p mimic 48 h transfection and G is the analysis of F. Data is expressed as mean ± standard deviation (analysis of variance, *p < 0.05, **p < 0.01, ***p < 0.001).

To further determine the role of miR-340-3p in the inhibition of PDFS cell growth by SZ-685C, PDFS cells were transfected with 10 nM miR-340-3p mimic, with the aim of exogenously increasing the expression of miR-340-3p in the control group and SZ-685C-treated group. Firstly, the transfection time and efficiency of miR-340-3p mimic were explored. As shown in Fig. 6A, compared with the control group, the highest expression of miR-340-3p was observed in PDFS cells after 48 h of miR-340-3p mimic transfection. Therefore, 48 h was used as the transfection time in subsequent transfection experiments.

In the qPCR experiment results (Fig. 6B), the overexpression of miR-340-3p significantly increased the expression of miR-340-3p in the Control + mimic group and SZ-685C + mimic group compared to the non-transfected group. However, the expression of miR-340-3p in the SZ-685C + mimic group decreased compared to the Control + mimic group. There was no significant difference in the expression of miR-340-3p between the Control + NC group, the SZ-685C + NC group, and the non-transfected group, indicating that miR-340-3p mimic transfection can effectively increase the expression of miR-340-3p in PDFS cells, while miR-340-3p NC transfection has the same effect as the non-transfected group.

To investigate whether the inhibitory effect of SZ-685C on PDFS cells changes after overexpression of miR-340-3p, a CCK-8 assay was conducted after miR-340-3p mimic transfection. As shown in Fig. 6C, the inhibition rate of PDFS cells in the SZ-685C + mimic group after 24 h of drug treatment was lower than that in the SZ-685C group, and the inhibition rate of PDFS cells in the Control + mimic group was also lower than that in the Control group. However, there was no significant difference in the inhibition rate between the SZ-685C + NC group and the SZ-685C group, and there was no significant difference in the inhibition rate between the Control + NC group and the Control group.

To investigate whether the inhibitory migration and proliferation effects of SZ-685C on PDFS cells have changed after overexpression of miR-340-3p, the wound healing assay and clone formation assay were conducted after miR-340-3p mimic transfection. As shown in Fig. 6D, the scratch width change of PDFS cells in the SZ-685C + mimic group within 24 h was smaller than that in the SZ-685C + NC group, and the cell migration rate was higher than that in the SZ-685C + NC group. However, the scratch width change of PDFS cells in the Control + mimic group was smaller than that in the Control + NC group within 24 h, and the cell migration rate was higher than that in the latter group (Fig. 6E).

The results of the plate clone formation experiment are shown in Fig. 6F. The number of PDFS cell colonies in the SZ-685C + mimic group increased compared to the SZ-685C + NC group, and the number of PDFS cell colonies in the Control + mimic group increased compared to the Control + NC group (Fig. 6G). These results indicate that overexpression of miR-340-3p can enhance the migration and proliferation of PDFS cells and weaken the inhibitory effect of SZ-685C on cell migration and proliferation of PDFS cells.

### 3.6 Autophagy promoting effect of SZ-685C on PDFS cells reduced after overexpression of miR-340-3p

**Fig. 7:**
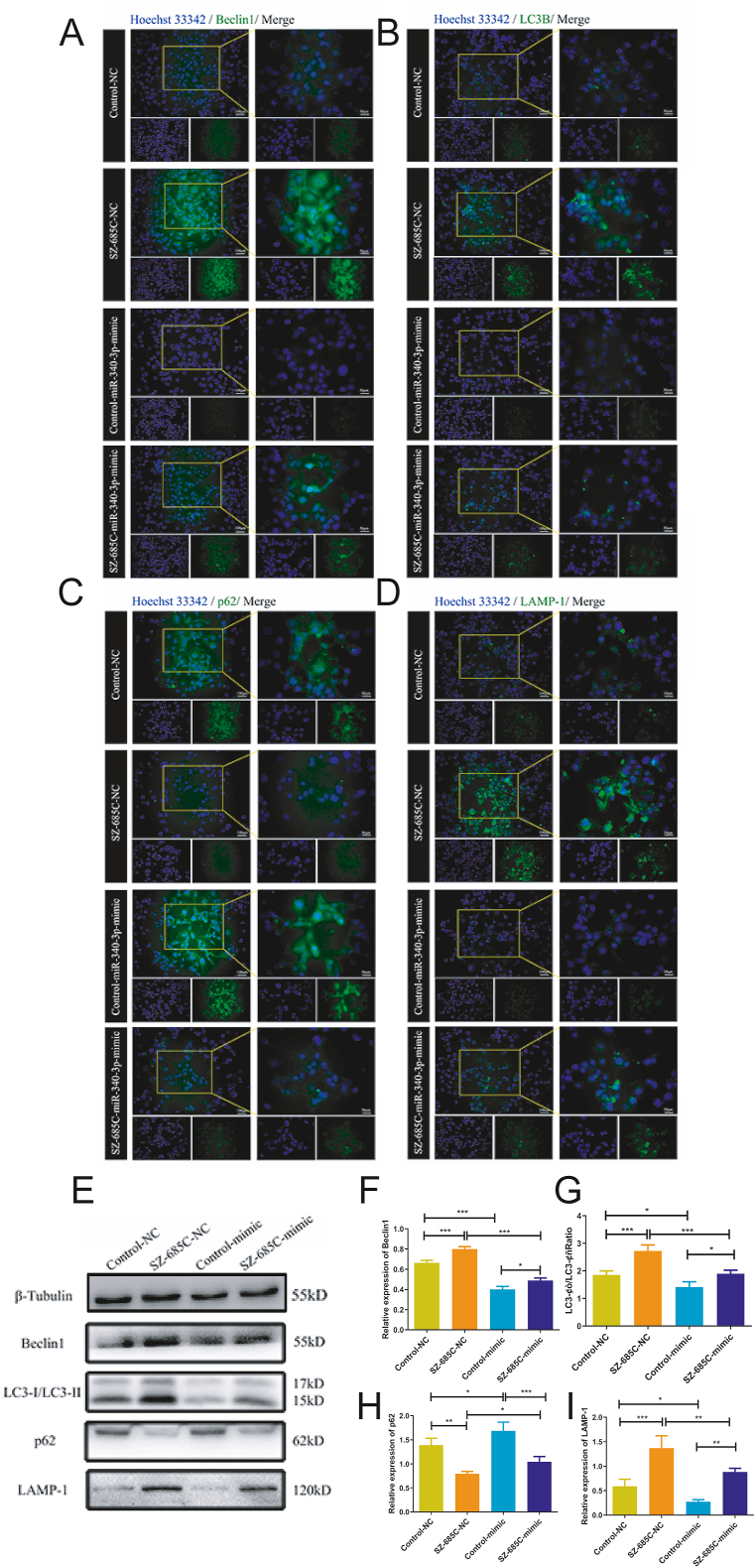
The effect of SZ-685C on autophagy-related protein of PDFS cells after miR-340-3p mimic transfection. A-D show the results of immunofluorescence assay including the expression levels of Beclin1, LC3B, p62 and LAMP-1 in each group, scale bar = 100 μm or 50 μm; E-I show the Western blot results of Beclin1, LC3-I, LC3-II, p62 and LAMP-1 in each group after miR-340-3p mimic transfection. (The original image is provided in Supplementary file) Data is expressed as mean ± standard deviation (analysis of variance, *p < 0.05, **p < 0.01, ***p < 0.001).

To investigate whether the autophagy promoting effect of SZ-685C on PDFS cells changes after overexpression of miR-340-3p, immunofluorescence and Western blot assays were conducted. The results of the immunofluorescence experiment are shown in Fig. 7A–D. Compared with the SZ-685C + NC group, the expression levels of Beclin1, LC3B, and LAMP-1 in the SZ-685C + mimic group decreased, while the expression level of p62 increased; Compared with the Control + NC group, the Control + mimic group showed a decrease in the expression levels of Beclin1, LC3B, and LAMP-1, while the expression level of p62 increased. As shown in Fig. 7E–I, Western blot results showed that the SZ-685C + mimic group had a lower expression level of Beclin1, a lower LC3-II/LC3-I ratio, an increase in p62 expression level, and a decrease in LAMP-1 expression level compared to the SZ-685C + NC group; Compared with the Control + NC group, the protein expression levels in the Control + mimic group decreased in Beclin1, LC3-II/LC3-I ratio decreased, p62 expression level increased, and LAMP-1 expression level decreased. Based on the above results, it is suggested that overexpression of miR-340-3p weakens the autophagy promoting effect of SZ-685C on PDFS cells.

### 3.7 SZ-685C downregulates miR-340-3p to reduce PPP1CB expression in PDFS cells

**Fig. 8:**
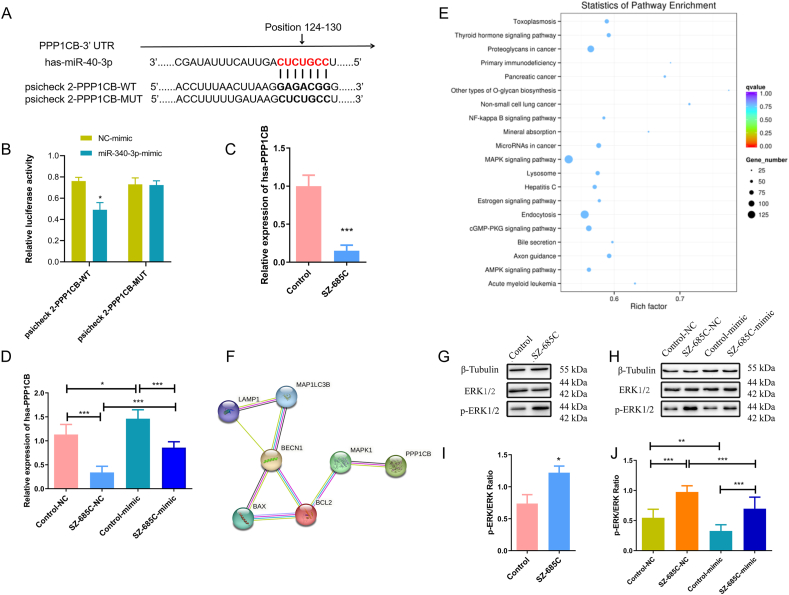
SZ-685C downregulates miR-340-3p to reduce PPP1CB and increase ERK 1/2 expression in PDFS cells. A shows the targeted binding region of miR-340-3p and PPP1CB 3′-UTR; B shows a dual-luciferase activity in PDFS cells co-transfected with NC mimic/miR-340-3p mimic and psicheck2-PPP1CB-WT/psicheck2-PPP1CB-MUT; C shows the expression level of PPP1CB mRNA in PDFS cells after SZ-685C treatment; D shows the expression level of PPP1CB mRNA in PDFS cells after miR-340-3p mimic transfection; E shows the result of KEGG pathway enrichment analysis; F shows the protein interaction network analysis; G shows the expressions of p-ERK1/2 and ERK1/2 proteins in PDFS cells after SZ-685C treatment and I is the analysis of G; H shows the expressions of p-ERK1/2 and ERK1/2 proteins in PDFS cells after miR-340-3p mimic transfection and J is the analysis of H. (The original image is provided in Supplementary file) Data is expressed as mean ± standard deviation (analysis of variance, *p < 0.05, **p < 0.01, ***p < 0.001).

miRDB and TargetScan analysis predicted one binding site in the 3′UTR of PPP1CB, suggesting that miR-340-3p may directly target PPP1CB (Fig. 8A). To confirm that PPP1CB is a direct target of miR-340-3p in PDFS cells, we co-transfected PDFS cells with miR-340-3p mimics and the PPP1CB reporter plasmid with either the WT or, as a control, the mutant type (MUT) 3′-UTR. As a control, we co-transfected PDFS cells with the NC mimics and the PPP1CB reporter plasmid containing either the WT or the MUT 3′-UTR. Then, we examined the dual-luciferase expression. We observed a greater reduction in the level of dual-luciferase expression in the WT group than in the NC or the MUT groups (Fig. 8B). Therefore, we concluded that miR-340-3p may target PPP1CB in PDFS cells.

In order to further investigate whether miR-340-3p regulates PPP1CB during the inhibition process of SZ-685C on PDFS cells, the expression of PPP1CB in PDFS cells after SZ-685C treatment was firstly detected. As shown in Fig. 8C, the expression level of PPP1CB mRNA in PDFS cells in the SZ-685C treated group was lower than that in the Control group. To determine whether miR-340-3p is related to the regulatory effect of PPP1CB, this chapter retested the expression of PPP1CB in PDFS cells after miR-340-3p mimic transfection. As shown in Fig. 8D, compared with the SZ-685C + NC group, the expression level of PPP1CB mRNA in the SZ-685C + mimic group increased; the expression level of PPP1CB mRNA in the Control + mimic group increased compared to the Control + NC group. The above results suggest that SZ-685C may reduce the expression of PPP1CB by downregulating miR-340-3p.

### 3.8 SZ-685C downregulates miR-340-3p to activate the ERK pathway in PDFS cells

Using the transcriptome sequencing results to conduct KEGG pathway enrichment analysis, the results show that the mechanism of SZ-685C may be closely related to the MAPK pathway (Fig. 8E). Through protein interaction network analysis, it was found that there may be an interaction relationship between PPP1CB and ERK proteins (Fig. 8F). To further investigate whether changes in the ERK pathway are involved in the inhibition process of SZ-685C on PDFS cells, the expression level of ERK1/2 protein and its phosphorylation form in PDFS cells treated with SZ-685C were evaluated through Western blot assay. As shown in Fig. 8G, compared with the Control group, the p-ERK/ERK ratio in PDFS cells treated with SZ-685C increased (Fig. 8H), indicating that the ERK pathway in PDFS cells may be activated after SZ-685C treatment. In order to investigate whether miR-340-3p is related to the regulation of the ERK pathway, this chapter retested the expression of ERK protein in PDFS cells treated with SZ-685C after overexpressing miR-340-3p. As shown in Fig. 8H, compared with the SZ-685C + NC group, the p-ERK/ERK ratio in the SZ-685C + mimic group decreased; Compared with the Control + NC group, the p-ERK/ERK ratio in the Control + mimic group decreased (Fig. 8J). The above results suggest that SZ-685C may promote the activation of the ERK signaling pathway by downregulating miR-340-3p.

## 4 Discussion

NFPA is a clinically refractory tumor and currently does not have FDA approved treatment drugs [22]. Due to the insidious clinical manifestations of NFPA and its low sensitivity to commonly used drugs for treating other types of pituitary adenomas, as well as the increased difficulty of surgery, frequent postoperative complications, and high risk of recurrence, the demand for drug development in NFPA has become more urgent [6]. It is important to note that many NFPA-related researches were difficult to carry out in the past due to a lack of human NFPA cell lines. The emergence of PDFS cell lines, which we used in this study, may be a good solution to this issue [23].

Anthracyclines have potent anticancer properties, making them a popular search term [24]. Some anthracyclines have been utilized in clinical trials, similar to doxorubicin and daunorubicin [25]. One of the outstanding examples of anthracycline anti-cancer compounds is SZ-685C. It has a plentiful source, isolated streptomyces from widely dispersed mangroves, so it has promising development potential. In the prior study, SZ-685C was discovered to inhibit the Akt pathway in vitro, leading to the induction of apoptosis in primary human NFPA cells [10]. In the current study, we showed that the inhibitory effect of SZ-685C on PDFS cells may result from autophagic cell death. This was shown by the altered levels of autophagy signature proteins, such as up-regulated LC3-II and Beclin1, as well as down-regulated p62, which indicates autophagy activation. SZ-685C has the ability to kill PDFS cells, making it a potentially effective method for treating NFPA.

One of the significant subcategories of cell death, which includes apoptosis, autophagy, ferroptosis, pyroptosis, and necroptosis, is regulated cell death (RCD) [26]. In some circumstances, such as during drug treatment, excessive autophagy can result in cell death [27]. Due to the discovery of a Beclin1 deficiency, autophagy is first endowed with oncogenic effects [28]. Studies have shown that activation of autophagy limits the growth and biological activity of tumors [[29], [30], [31]]. Celastrol, rosiglitazone, ACT001, and other prospective therapeutic medicines were found to exhibit anti-tumor activities linked to triggered autophagy cell death in studies of the molecular mechanisms of therapies for the treatment of PA [[32], [33], [34]]. Similar to this, we speculate that SZ-685C exerts anti-NFPA effect by triggering autophagic cell death.

Finding other abnormal factors in the body of NFPA patients can provide significant assistance in the diagnosis and treatment of tumors. Research has shown that many biological processes in cells involve changes in miRNAs, and their frequent involvement in various tumor processes, including invasion and treatment responses [35]. MiRNA regulates the expression sequence of post-transcriptional protein coding genes by targeting the UTR region of mRNA to form base pairs, making it play an important role in regulating various proteins, including oncoproteins and tumor suppressor proteins, and its imbalance can disrupt the balance of biological processes in the body and potentially lead to tumor development. For example, Zhu Zhang et al. found that miRNA-149 dependent downregulation of ADAM12 and MMP-14 expression in PA cells inhibits cell proliferation and invasion [36]. Q Xu et al. found that miR-137 can target AKT2 and inhibit AKT2 induced MMQ cell proliferation [37].

MiRNA has been shown to regulate autophagy by influencing related proteins that play a regulatory role in different stages of autophagy. In some cases, abnormal activation of autophagy associated with changes in miRNA levels is harmful to tumor cells themselves and simultaneously leads to cell apoptosis, autophagic cell death, or necrotic death [20]. For example, Banzhou Pan et al. found that in advanced small cell lung cancer, downregulation of miR-24–3p increased autophagy levels, directly targeting and inhibiting the ATG4A gene, suggesting that regulating miR-24–3p and autophagy may be the strategic basis for combined chemotherapy to prevent and treat lung cancer [38]. In this study, differential miRNAs of PDFS cells in the Control group and SZ-685C treatment group were detected by transcriptome sequencing, and miR-340-3p was specifically downregulated after SZ-685C treatment. Through in-depth exploration of the regulatory role of miR-340-3p, it was found that overexpression of miR-340-3p can weaken the inhibitory ability of SZ-685C on PDFS cells, including the cell viability, migration ability, proliferation ability, and the activation of autophagy, indicating that miR-340-3p may be involved in the autophagic cell death process of SZ-685C regulating PDFS cells.

Protein phosphorylation is one of the most common post translational modifications in eukaryotes. Among them, protein phosphatase PP1 is a serine/threonine specific protein phosphatase that catalyzes most eukaryotic protein dephosphorylation reactions in a selectively highly regulated manner, participating in multiple processes such as cell survival, cell cycle regulation, and apoptosis [39]. As one of the catalytic subunits of PP1, PPP1CB has been found to be critical to the dephosphorylation of myosin light chain [40], frequently involved in regulating the cytoskeleton network and the process of cell migration [41], and closely related to tumor invasion and metastasis [42]. In this study, miRDB and TargetScan analysis predicted one binding site in the 3′UTR of PPP1CB, suggesting that miR-340-3p may directly target PPP1CB. Furthermore, dual-luciferase assay confirmed the direct binding between miR-340-3p and PPP1CB. Combined with the qRT-PCR experimental results, it was preliminarily concluded that the downregulation of miR-340-3p in PDFS cells may be accompanied by a decrease in PPP1CB expression. The above suggests that miR-340-3p and PPP1CB may have the same trend of change in PDFS cells.

Previous studies have shown that miRNAs can inhibit the translation process by binding to the 3′-UTR of the target gene, thereby cutting mRNA and inhibiting its expression [43]. However, existing evidence suggests that miRNAs can also upregulate gene expression after transcription [44,45], and can even protect mRNA from being cleaved by other miRNAs by binding to the 3′-UTR region [43]. The binding process has the characteristics of selectivity and partial regulation, and whether the effect of miRNA on mRNA is up-regulated or down-regulated depends on the RNA sequence background, complexity of the regulatory process, and specific cell state [44]. The results of this study indicate that miR-340-3p has a positive regulatory effect on PPP1CB, indicating that there may be a direct targeted upregulation mechanism between miR-340-3p and PPP1CB, and there may also be an intermediate target between miR-340-3p and PPP1CB, allowing miR-340-3p to indirectly exert a positive regulatory effect on PPP1CB through this intermediate target.

In this study, protein interaction network analysis was used to predict the interaction relationship between PPP1CB and ERK protein, and the experimental results suggest that the downregulation of PPP1CB is accompanied by the activation of ERK pathway in PDFS cells treated with SZ-685C. Florian P ö ll et al. found that in human embryonic kidney HEK293 cells, a decrease in PPP1CB activity resulted in an abnormal increase in somatostatin and substance P-induced ERK pathway activation [46], indicating a connection between PPP1CB and ERK protein. Combining the previous two chapters, it is suggested that SZ-685C may inhibit the growth of PDFS cells by downregulating miR-340-3p, regulating the PPP1CB/ERK pathway, promoting autophagic cell death. ERK/MAPK pathway is one of the main altered molecular pathways in NFPA [47], and several studies demonstrated that ERK activation is significantly associated with autophagic cell death [48]. For illustration, soyasaponins have been discovered to induce macroautophagy in human colon cancer cells via enhancing ERK1/2 activation [49]. Additionally, treatment with ubenimex could cause autophagic cell death by activating the ROS/ERK pathway in rat PA cell lines GH3 and MMQ [50]. The involvement of ERK in PDFS cells suggests that ERK is a key target for the development of anti-NFPA medications. This study only explored the role of SZ-685C in vitro but not in vivo, which has certain limitations, resulting in the depth of the study is enough but the breadth is not enough, which to some extent affects the comprehensiveness of the results, so in the future we will explore in depth the more comprehensive antitumor mechanism of SZ-685C.

## 5 Conclusion

Above all, the results of this study suggest that SZ-685C may downregulate the expression of miR-340-3p in PDFS cells, thereby reducing the expression of PPP1CB and activating the ERK pathway to promote autophagic cell death, thereby inhibiting cell growth.

## Funding

This study was supported by the 10.13039/501100001809National Natural Science Foundation of China for Young Scholars [grant number 81802678]; the 10.13039/501100003453Guangdong Natural Science Foundation Project [grant number 2018A030310107]; the Guangzhou Basic and Applied Basic Research Foundation [grant number 202102021116], [grant number 202201010307]; Key Clinical Technique of Guangzhou (2023P-ZD18); the Guangdong Province Administration of Traditional Chinese Medicine Project [grant number 20231210].

## Institutional review board statement

Not applicable.

## Informed consent statement

Not applicable.

## Data availability statement

The original contributions presented in the study are included in the article/Supplementary Material, further inquiries can be directed to the corresponding author/s.

## CRediT authorship contribution statement

**Xin Wang:** Writing – original draft, Funding acquisition, Conceptualization. **Zhong-Yu Wang:** Writing – original draft. **Hui-Tong Chen:** Data curation. **Yu-You Luo:** Data curation. **Si-Yuan Li:** Data curation. **Xiong-Ming Luo:** Visualization, Supervision. **Jun-Hua Yang:** Visualization, Supervision. **Yu-Xin Ma:** Visualization, Supervision. **Xiao-Bao Jin:** Visualization, Supervision. **Jing Liu:** Writing – review & editing, Project administration. **Zong-Ming Wang:** Writing – review & editing, Project administration.

## Declaration of competing interest

The authors declare that they have no known competing financial interests or personal relationships that could have appeared to influence the work reported in this paper.
