# Supplementary material for: SZ-685C inhibits the growth of non-functioning pituitary adenoma by down-regulating miR-340-3p and inducing autophagy
Source: Heliyon. 2024 Aug 30;10(17):e37230. doi: 10.1016/j.heliyon.2024.e37230 (PMC11402753; doi:10.1016/j.heliyon.2024.e37230)

Fig-4E
β-Tubulin

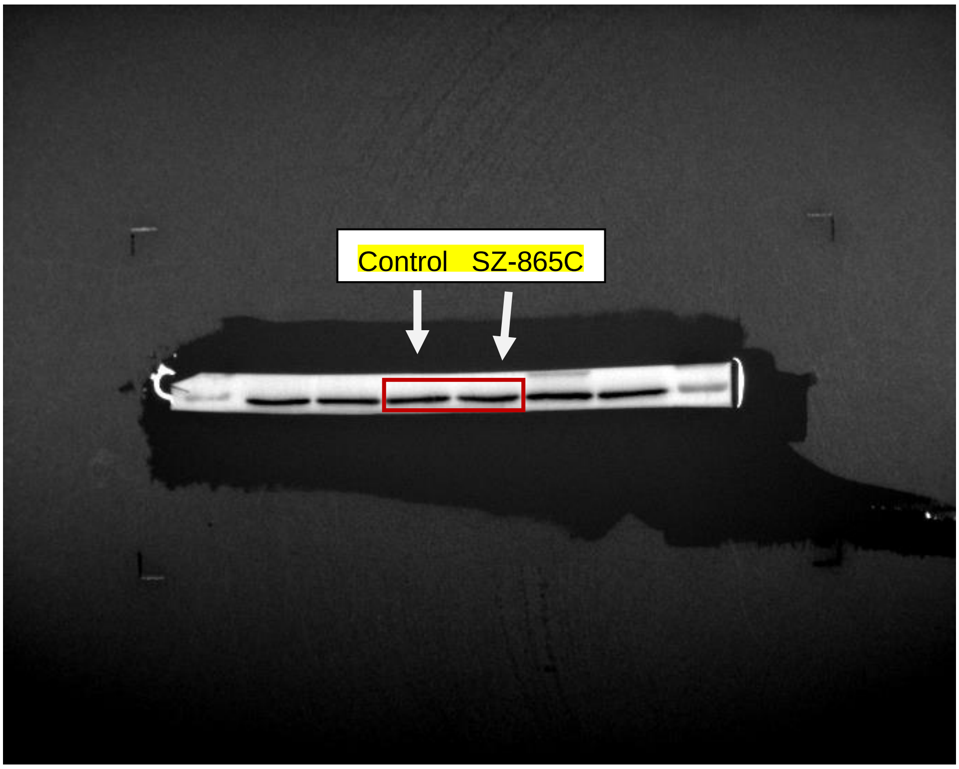

Fig-4E
Beclin1

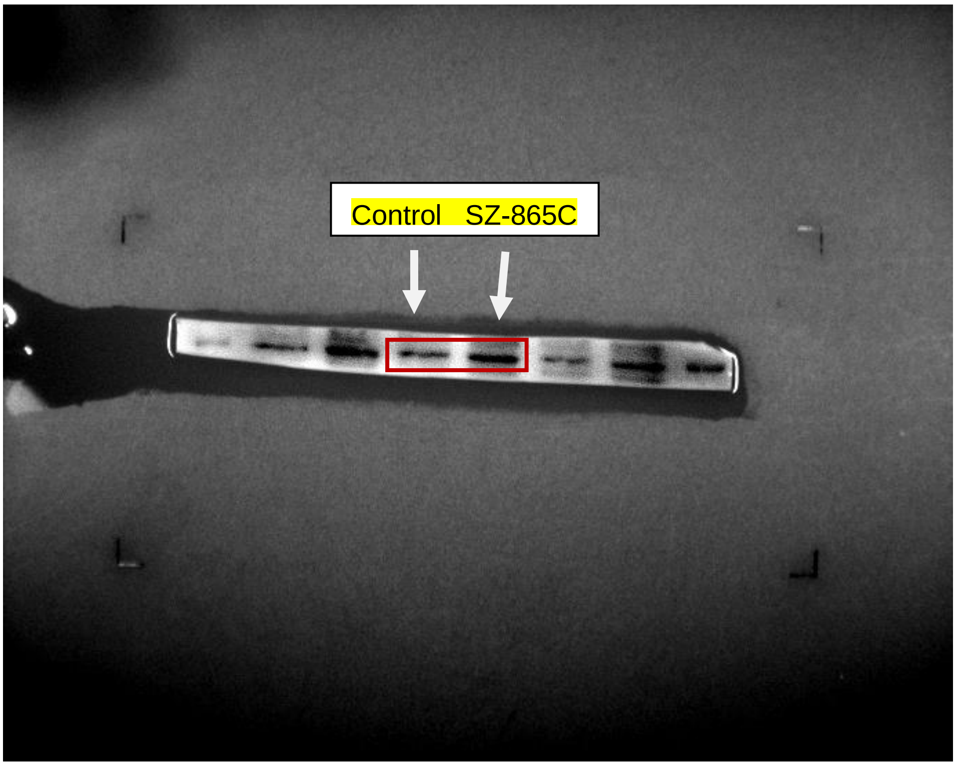

Fig-4E
LC3

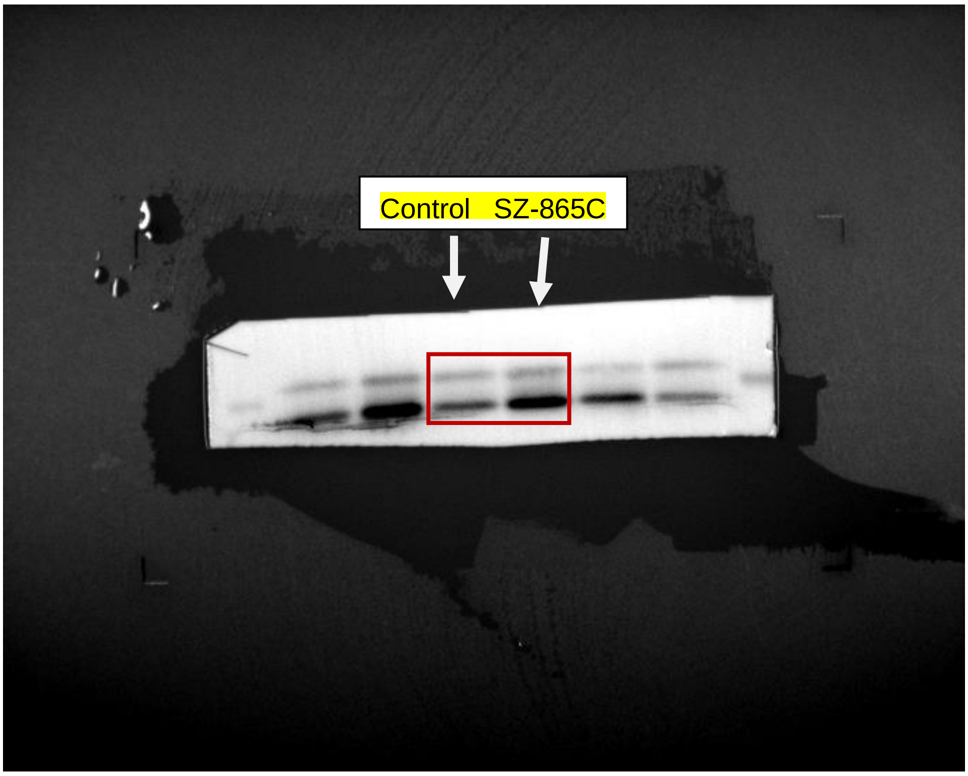

Fig-4E
P62

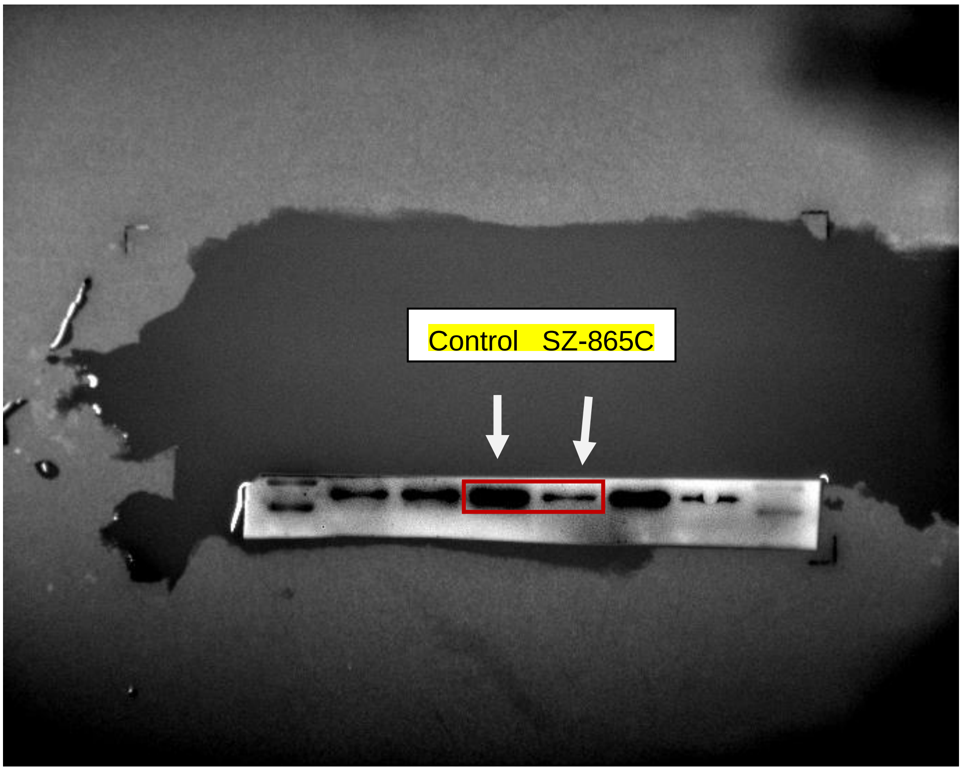

Fig-4E
LAMP-1

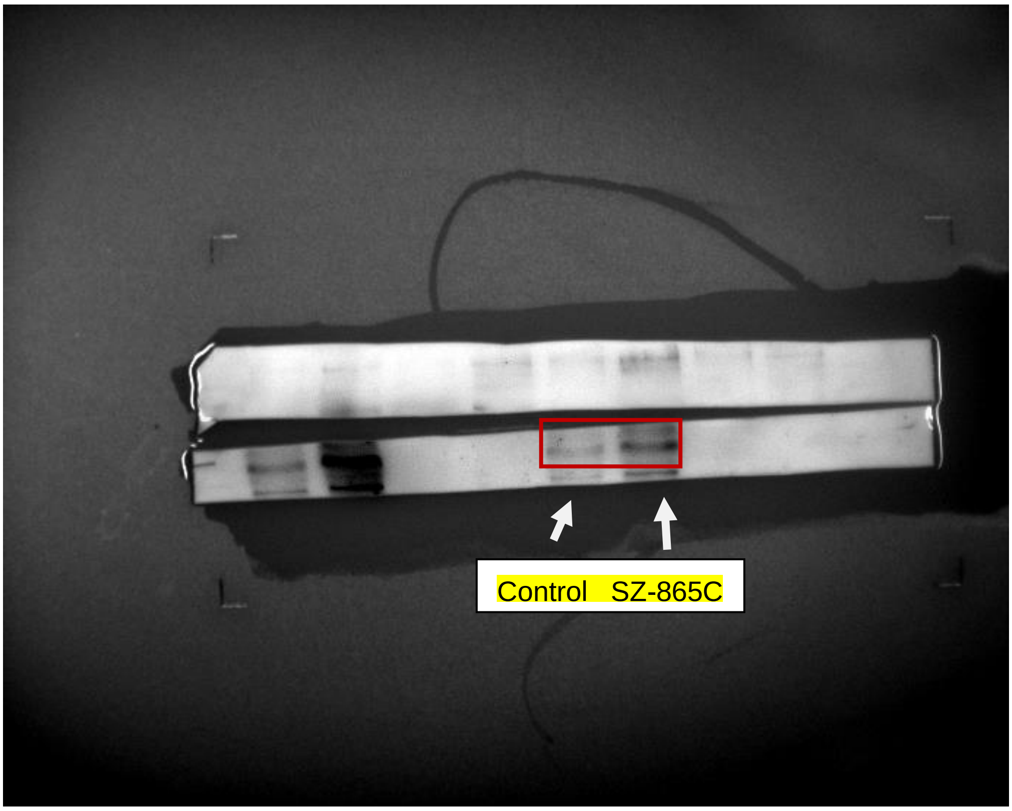

Fig-7E
β-Tubulin

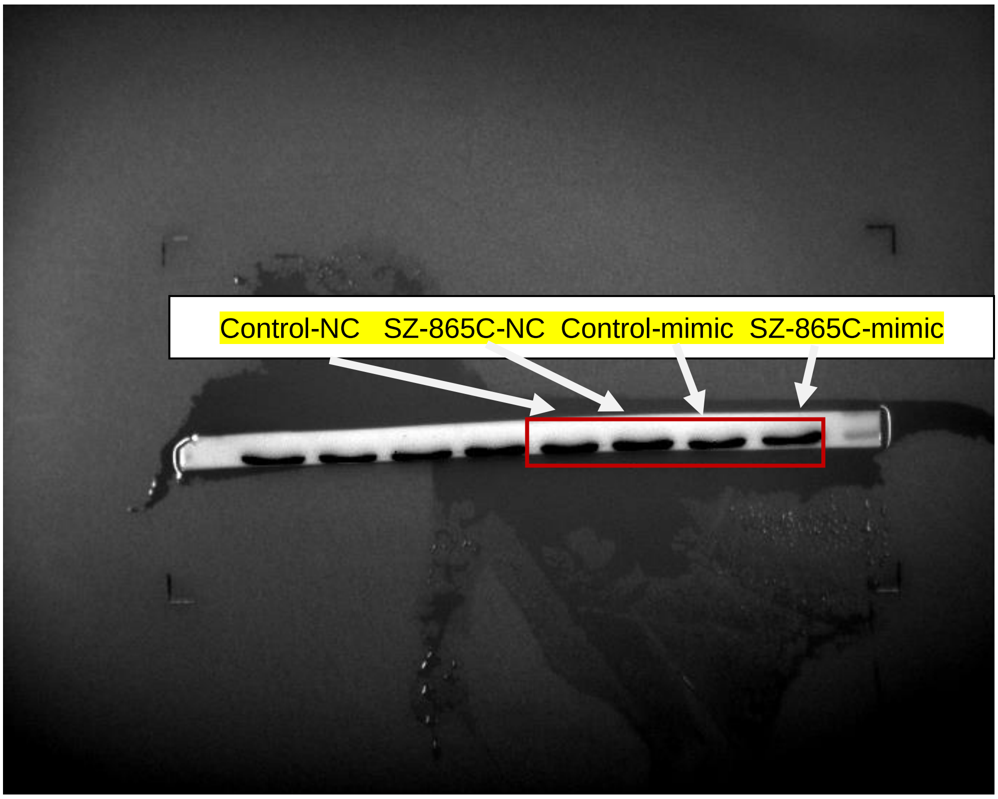

Fig-7E
Beclin1

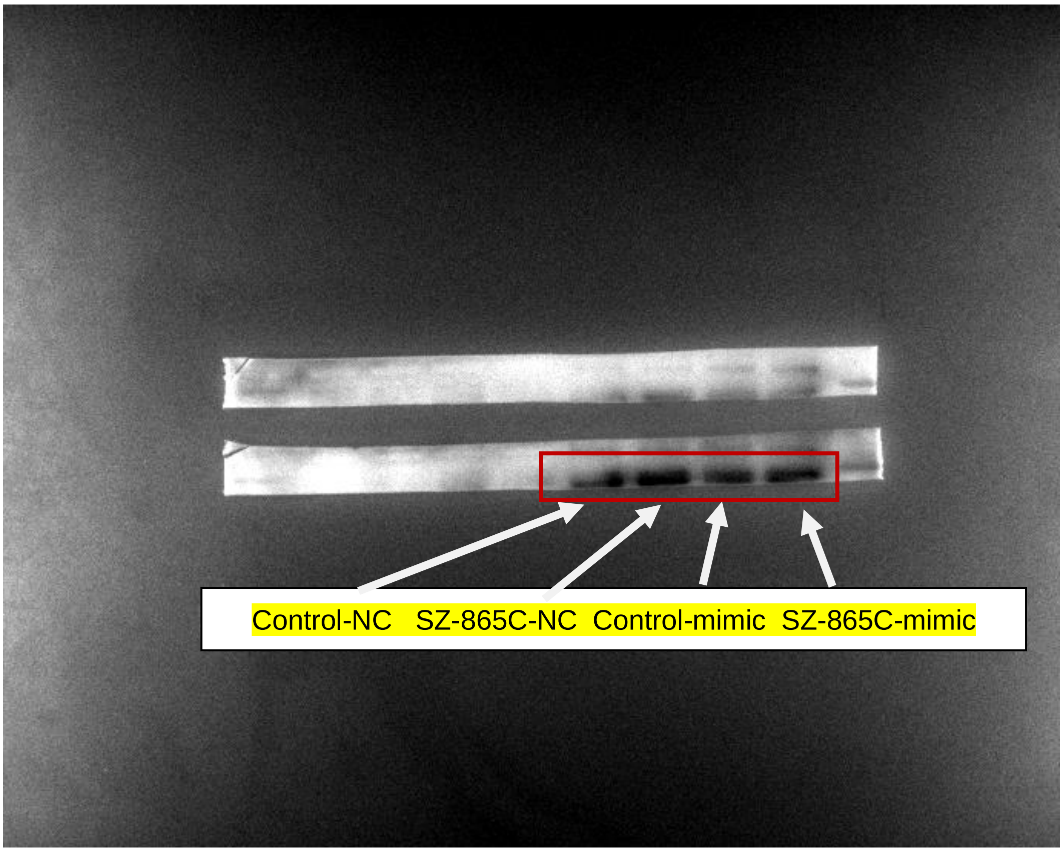

Fig-7E
LC3

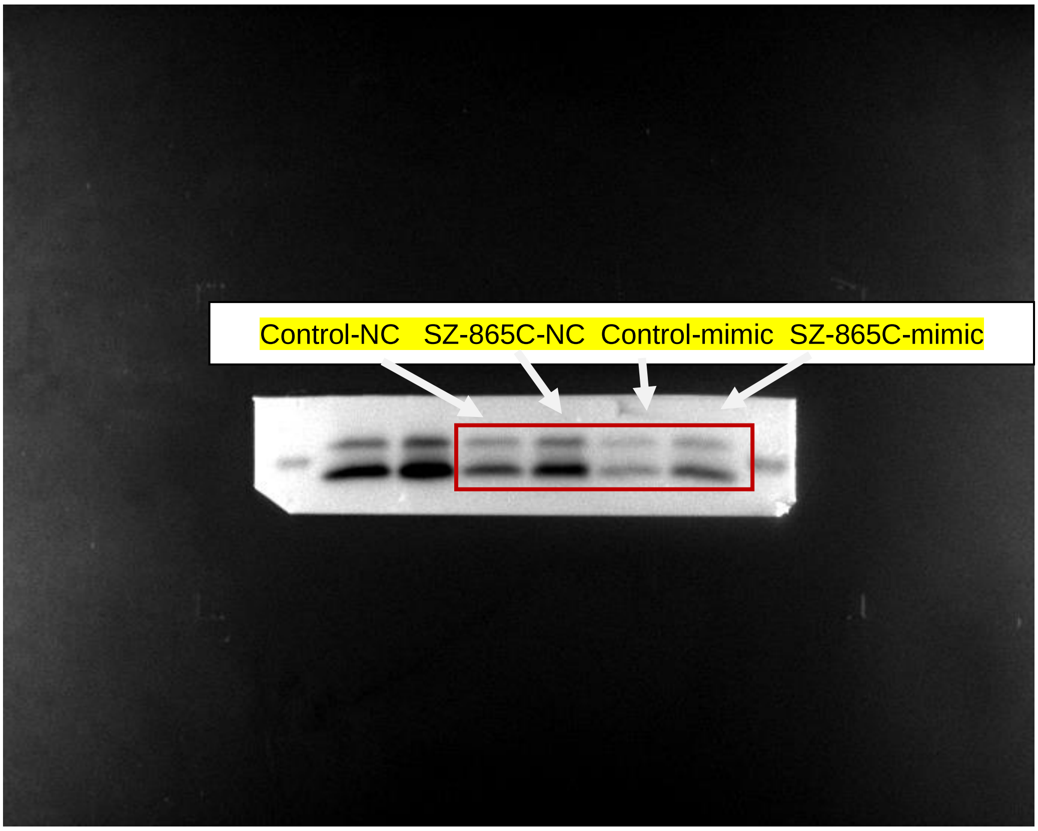

Fig-7E
P62

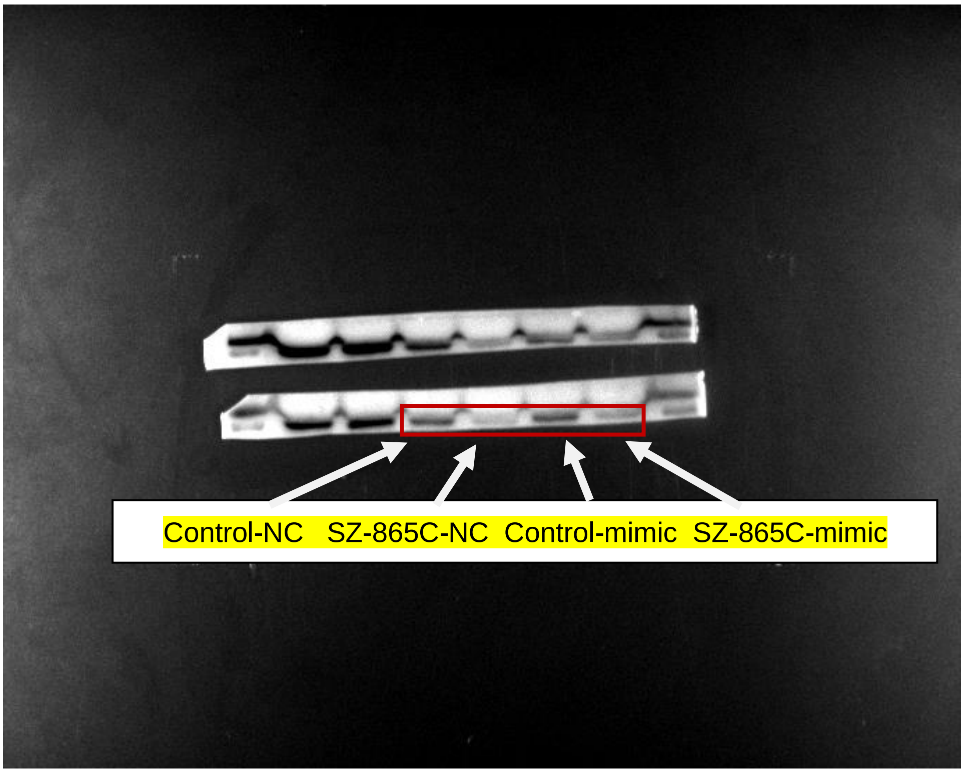

Fig-7E
LAMP-1

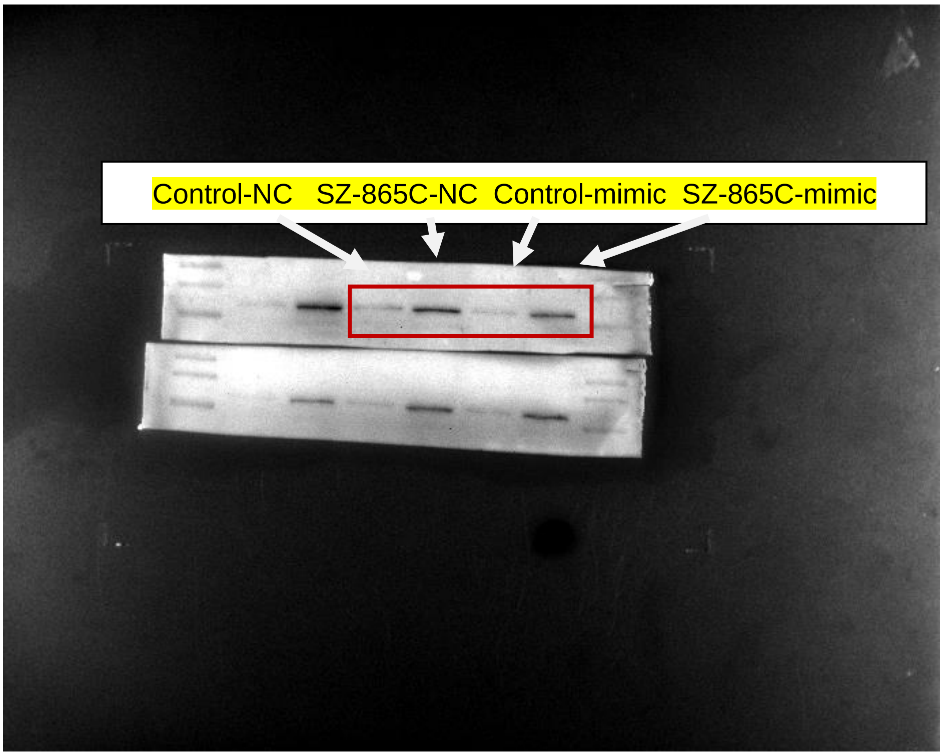

Fig-8G
β-Tubulin

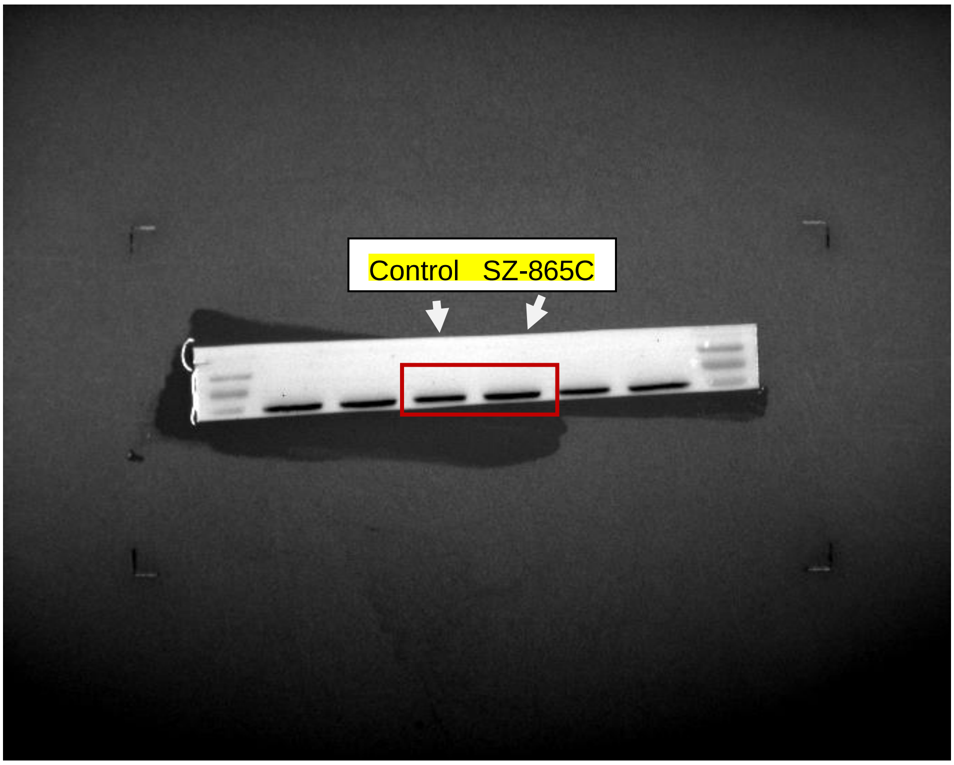

Fig-8G
ERK 1/2

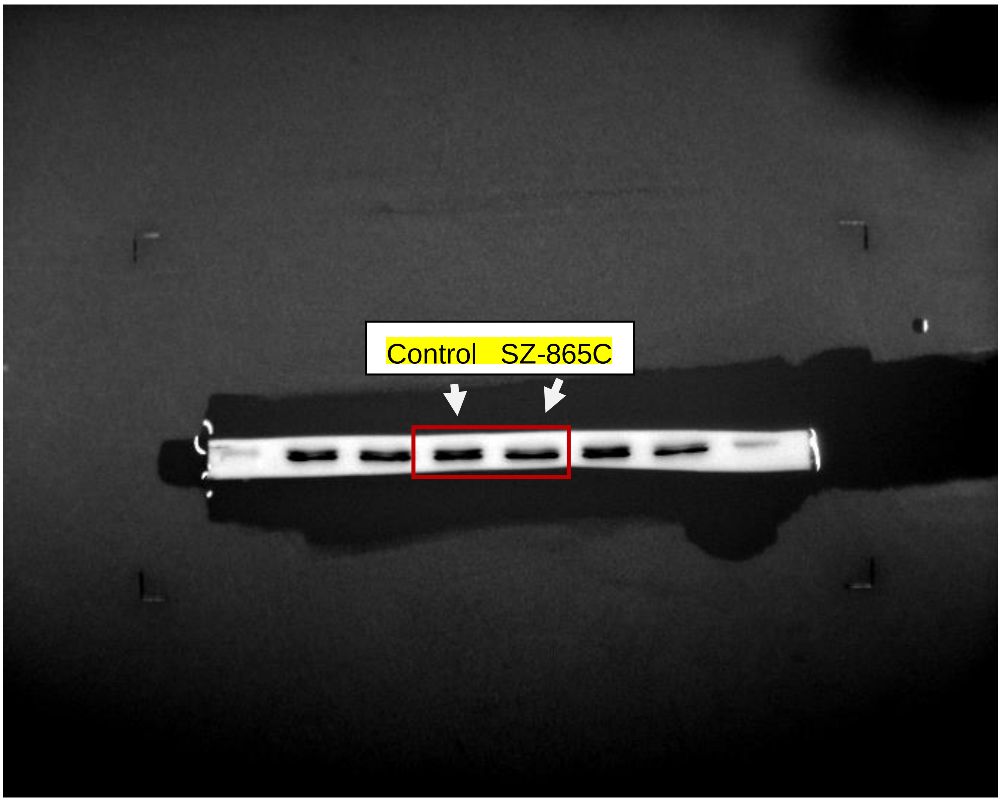

Fig-8G
p-ERK 1/2

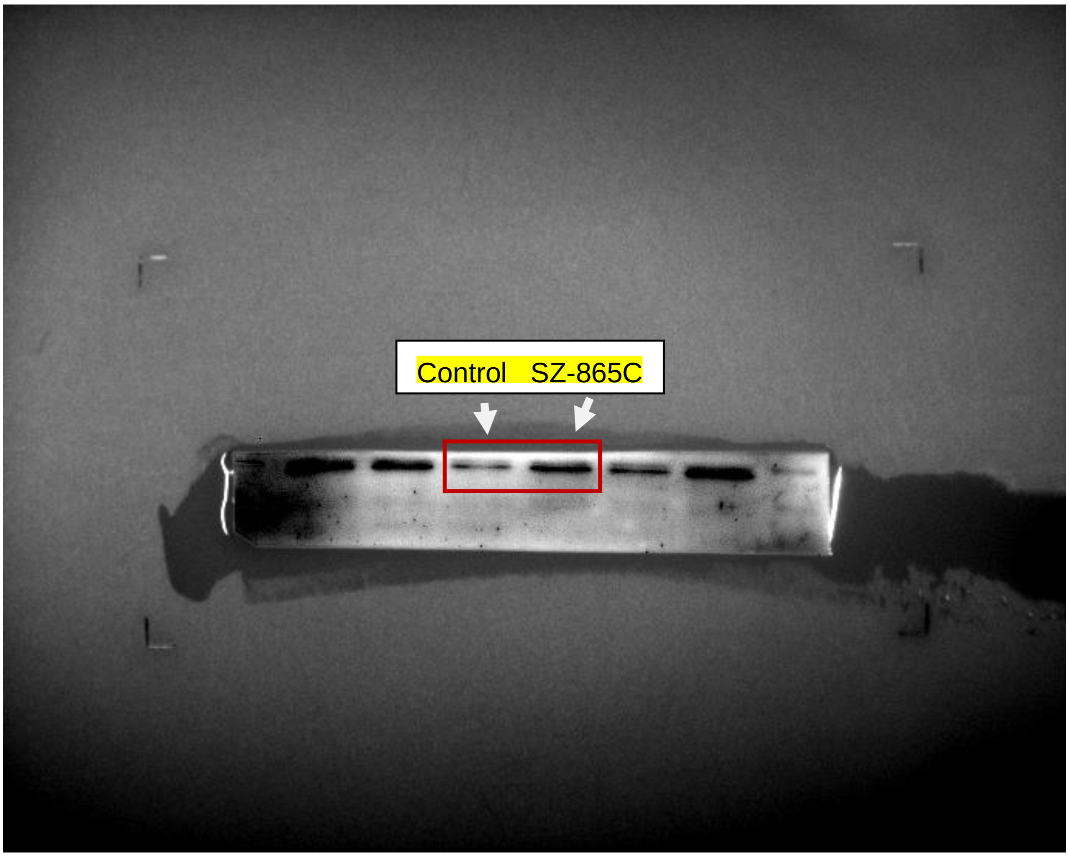

Fig-8H
β-Tubulin

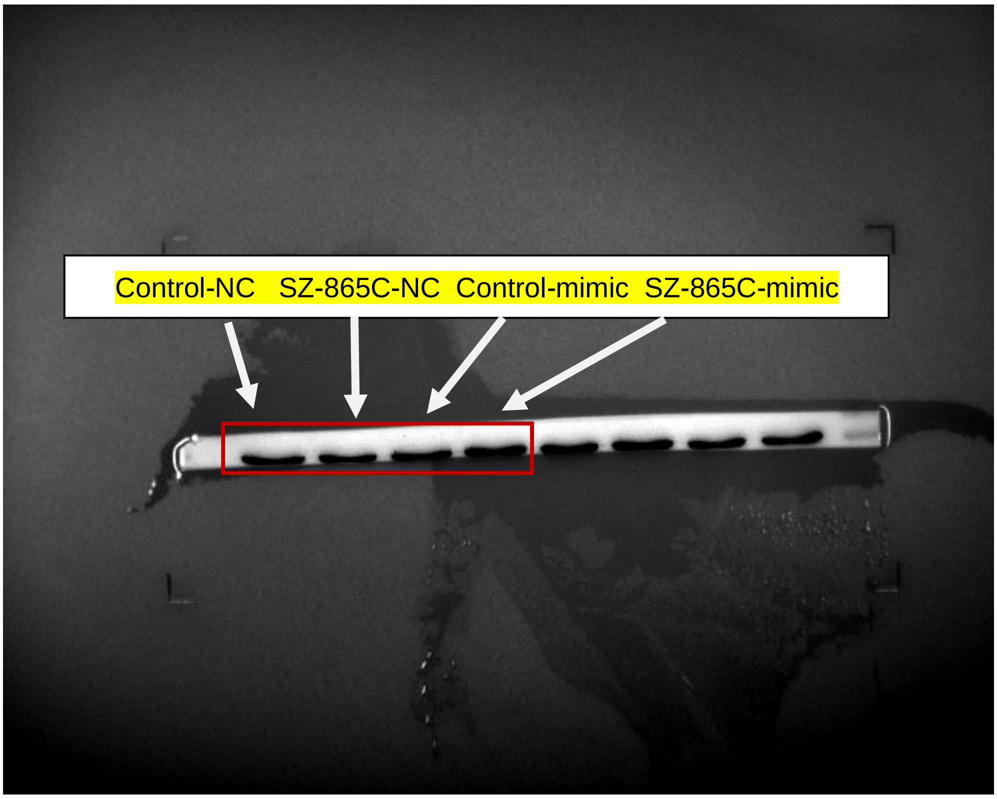

Fig-8H
ERK 1/2

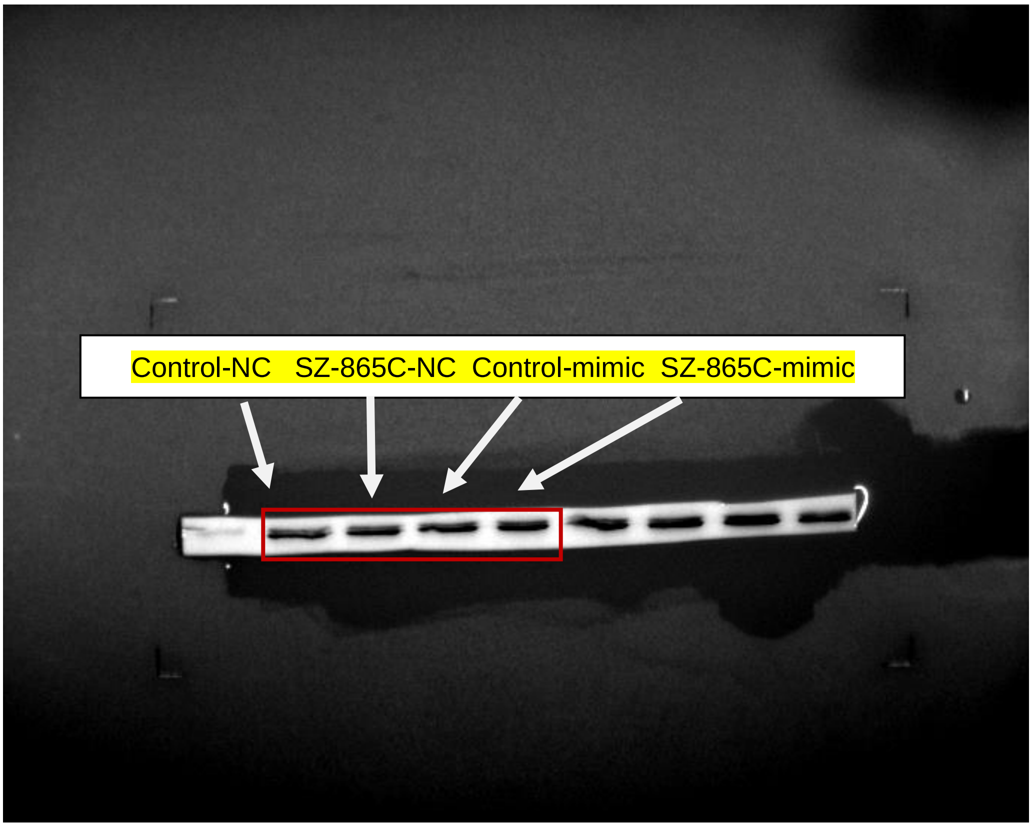

Fig-8H
p-ERK 1/2

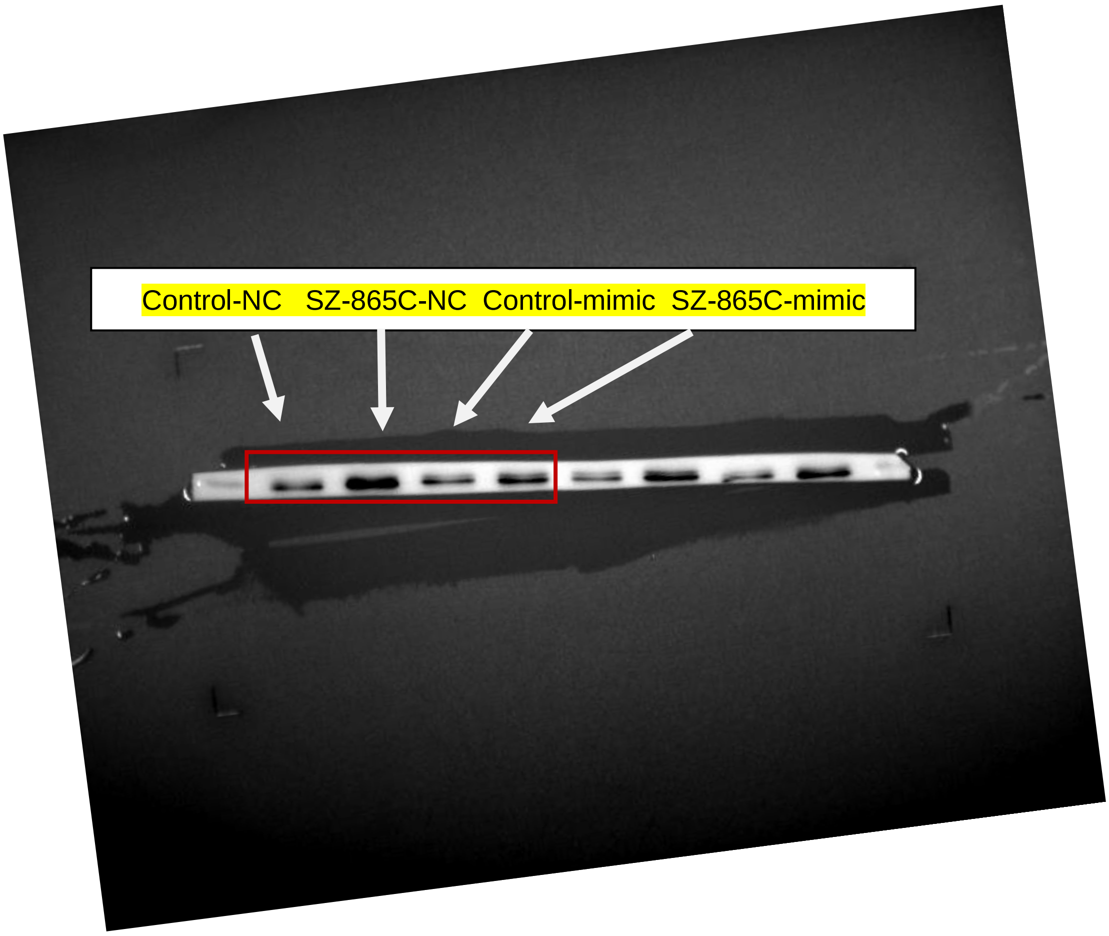

Supplement: Multimedia component 1 [file mmc1.pdf]
